# Effect of Reduction in Dietary Amino Acids and Energy on Growth Performance and Economic Return of Cobb 700 and Ross 708 Broilers

**DOI:** 10.3390/ani15060890

**Published:** 2025-03-20

**Authors:** Bo Zhang, Shengyu Zhou, Wei Zhai, Yang Zhao

**Affiliations:** 1 The Department of Poultry Science, Mississippi State University, Starkville, MS 39762, USA; bozhmsu@gmail.com (B.Z.); wei.zhai@msstate.edu (W.Z.); 2 The Department of Animal Science, The University of Tennessee, Knoxville, TN 37996, USA; zhoushengyuxnkl@gmail.com

**Keywords:** nutrient reduction, broiler, growth performance, marginal return, feed cost

## Abstract

This study examined how reducing dietary amino acids (AAs) and apparent metabolizable energy (AME) affects broiler growth and economic return. Two trials were conducted with Cobb 700 and Ross 708 broilers using a randomized design with 12 diet treatments. A 30% AA reduction, compared to the high breeder recommendations for Cobb 700 and Ross 708 strains, reduced body weight in Cobb 700 broilers before 41 days and in Ross 708 broilers at 64 days, while feed efficiency declined in both strains from 0 to 41 days. A 16% AME reduction increased feed intake and worsened feed efficiency in Cobb 700 broilers before 41 days and in Ross 708 broilers from 55 to 64 days. At 55 days, reducing AAs by 20–30% or AME by 8–16% decreased feed cost per kilogram of body weight and improved economic return. However, a 30% AA reduction reduced overall profitability per bird in Cobb 700 broilers at 41 days, while a 16% AME reduction did not. In conclusion, both 30% AA and 16% AME reductions impaired growth, but only excessive AA reduction negatively affected profitability. These findings help optimize feeding strategies for cost-effective broiler production.

## Simple Summary

Advances in genetics and nutrition over the past 70 years have greatly improved the growth rate and efficiency of modern commercial broilers. However, commercial-type diets designed for maximum growth often contain higher nutrient levels than economically necessary, leading to increased feed costs. While reducing dietary amino acids and energy can lower costs, their combined effects on broiler growth and profitability remain unclear. This study examined how decreasing amino acids and energy in broiler diets impacts growth performance and economic return in two broiler strains, Cobb 700 and Ross 708. The results showed that a 30% reduction in amino acids, compared to the high breeder recommendations for Cobb 700 and Ross 708 strains, led to lower body weight and poorer feed efficiency. In contrast, a 16% reduction in energy also negatively affected growth but had a smaller impact on profitability. At 55 days, both amino acid and energy reductions improved economic return per kilogram of body weight, though a 30% amino acids reduction lowered overall profitability per bird in Cobb 700 broilers. These findings suggest that moderate energy reduction may help manage feed costs without significantly harming economic returns, while excessive amino acid reduction may not be beneficial. This research helps poultry producers optimize feeding strategies, balancing growth performance with cost efficiency for sustainable and profitable production.

## 1. Introduction

Over the past seven decades, genetic selection and nutritional advancements have significantly improved the growth performance of modern broilers. Ross 308 broilers from 2005 grew at 104 g/day, nearly five times faster than the 1957 Alberta Meat Control strain. Their feed conversion ratio (FCR) also improved by 40%, dropping from 2.87 to 1.81 [1]. While nutritional advancements have helped maximize genetic potential [2,3,4], the focus on achieving maximum growth often requires high levels of dietary nutrients [4], which may not optimize economic returns. A previous study reported that the gross margin return was reduced with an increase in dietary energy from 3175 to 3265 kcal/kg [5]. Thus, commercial diets designed for maximum growth performance might include higher nutrient levels than economically necessary, suggesting that nutrient reduction could help control feed costs and improve profitability.

Dietary nutrient reduction has been suggested to decrease feed costs and influence the growth performance of broilers. Corzo et al. [6] reported that broilers fed a low-AA diet (85% of high-AA diet) exhibited a lower BW and a higher FCR as compared with broilers fed a high-AA diet during d 0–14. In the same study, the feed cost/BW was decreased by dietary AA reduction. Zhang et al. [7] also observed that reducing dietary digestible amino acids (lysine, total sulfur amino acids, and threonine) by 20% led to a decrease in feed cost per unit of body weight. In addition to dietary AA reduction, Dozier III et al. [5] found that the FCR of broilers linearly increased with the decreasing dietary energy from 3310 to 3175 kcal/kg. The marginal return was increased from 2.49 to 2.53 USD/bird when dietary energy decreased from 3265 to 3175 kcal/kg. These studies have suggested that dietary reduction in AAs and energy could be individually used to control feed costs.

There is still much to explore regarding the simultaneous reduction in amino acids (AAs) and energy in broiler diets. Yang et al. [8] demonstrated that body weight and FCR were significantly influenced by the interaction between protein and energy levels. Their study found that low-AME and high-AA diets reduced feed intake and BW at multiple time points (days 35, 42, and 54), likely due to metabolic constraints under heat stress, which subsequently led to lower carcass weight, breast weight, and wing yield. Conversely, high-AME and low-AA diets enhanced feed intake, BW gain, and feed conversion efficiency, ultimately increasing breast meat yield without additional feed costs. Further improvements in weight gain were observed with high-AME and high-AA diets. However, their study focused on total protein rather than digestible AA, which plays a more precise role in broiler nutrition. Therefore, the current study aimed to investigate the combined effects of reducing dietary AAs and energy on broiler growth and economic return, seeking to identify optimal nutrient reductions for better feed cost management and profitability.

## 2. Materials and Methods

### 2.1. Birds, Diets, and Management

All experiments followed the Guide for the Care and Use of Agriculture Animals in Research and Teaching [9] and the Mississippi State University Institution Animal Care and Use Committee, IACUC Animal Welfare Assurance #A3160-01. Trial 1 used Cobb 700 and Trial 2 used Ross 708 broilers. In each trial, a total of 864 day-old chicks were obtained from a local commercial hatchery. Ross 708 broilers were feather sexed, and Cobb 700 broilers were vent sexed upon arrival. The chicks were randomly distributed into 12 pens, each with 6 replicate blocks, in an environmentally controlled house. There were 6 male and 6 female broilers per pen (1.4 m^2^/pen, 0.117 m^2^/bird). Each pen was randomly assigned to 1 of the 12 dietary treatments (4 AA × 3 energy). The nutritional compositions of corn and soybean meal were obtained by using near-infrared spectroscopy (NIR, FOSS XDS, Denmark) before formulating the diets. Each trial included 12 dietary treatments with factorial combinations of four levels of amino acids (AAs) and three levels of apparent metabolizable energy (AME). Four levels of digestible AAs (Lys, total sulfur amino acids (TSAAs), and Thr) were formulated at 70%, 80%, 90%, and 100% of high breeder recommendations by Cobb 700 and Ross 708 strains [10,11]. Three AME levels were formulated at 84%, 92%, and 100% of high breeder recommendations for Cobb 700 and Ross 708 strains. In addition, sand was added to the feed as a filler to obtain 84% and 92% AME diets. These 12 diets were formulated in each of the four-phase programs, including Starter (d 0–10, Table 1), Grower (d 11–24, Table 2), Finisher (d 25–39, Table 3), and Withdrawal (d 40–63, Table 4). Feeds were provided in the form of crumbles during the Starter phase and pellets in the latter three phases. All broilers consumed feed and water on an ad libitum basis. Each pen was equipped with a hanging feeder and 3 nipple drinkers. The birds received a photoperiod of 24 h lightness and 0 h darkness from 0 to 7 d and a photoperiod of 20 h lightness and 4 h darkness from 8 to 64 d of age. Environment management, including ventilation, light intensity, and temperature, in the experimental room was adjusted according to the Ross 708 [12] and Cobb 700 [13] broiler management guides by bird age.

### 2.2. Growth Performance

In each trial, birds and feed were weighed by pen at 0, 10, 24, 34, 41, 48, 55, and 63 d of age. Daily BW gain (DBWG), daily feed intake (DFI), and feed conversion ratio (FCR) were calculated between each age period and the overall periods from 0 d of age. Daily mortality was recorded, and the weight of dead birds was accounted for in the calculation of FCR. Sand was added to the feed with 84% and 92% AME diets. The FCR and DFI without sand were also calculated. The growth rate (GR) was calculated by dividing body weight gain (BWG) by the initial BW in each age interval [7].

### 2.3. Economic Analysis

#### 2.3.1. Cost of Feed per Unit of Body Weight (BW)

At 41 and 55 d of age, the feed cost/BW was calculated following the method by Zhang et al. [7].

Feed cost/BW on d 41 = (Starter feed price × Starter FI + Grower feed price × Grower FI + Finisher feed price × Finisher FI)/BW on d 41(1)

Feed cost/BW on d 55 = (Starter feed price × Starter FI + Grower feed price × Grower FI + Finisher feed price × Finisher FI + Withdrawal feed price × Withdrawal FI)/BW on d 55(2)

#### 2.3.2. Gross Margin Return

At 41 and 55 d of age, the gross margin return (GMR)/bird and GMR/BW were calculated following the method by Zhang et al. [7].

Gross margin return/bird = BW × whole body price* − feeding cost(3)

Gross margin return/kg of bird = (BW × whole chicken price* − feeding cost)/BW(4)

* The whole chicken price was USD 1.809/kg at the time of calculation on 6 December 2019 [14].

### 2.4. Statistical Analysis

A randomized complete block design with a factorial arrangement of 12 treatments (4 AA × 3 AME) was used in each trial. The pen was considered the experimental unit. Dietary AAs and AME were considered as fixed effects, and the block was considered as a random effect in the model. The ratio of male to female broilers was regarded as a covariance factor in the analysis. The results of BW, DBWG, DFI, FCR, mortality, feed cost, and gross marginal return were analyzed by using a two-way ANOVA of a PROC GLM procedure of SAS version 9.4. Means were considered significantly different at *p* < 0.05 and were separated by using the Tukey–Kramer comparison test.

## 3. Results

The means and *p*-values of the main effects and interactive effects of AAs and AME are provided in each table. Only significant responses are noted in the Results Section. When the interaction is significant for a variable, the main effects means are not included in this section.

### 3.1. Trial 1 of Cobb 700 Broilers

#### 3.1.1. Body Weight and Daily Body Weight Gain

The interaction between AAs and AME significantly affected BW. Broilers fed 70% AAs with 100% AME exhibited lower BW (2631 g) compared to those fed 100% AAs with 84% AME (3217 g) at day 48 (*p* = 0.02, Table 5). This difference continued through day 55, with the 70% AA, 100% AME group weighing 2977 g, while the 100% AA, 84% AME group reached 3665 g (*p* = 0.01).

Cobb 700 broilers fed 70% AAs exhibited the lowest BW among all levels of dietary AAs at 10 and 24 d of age (240.8 g and 899 g, respectively, *p* < 0.01, and <0.01). On day 34, broilers fed 70% AAs continued to show lower BW (1634 g) compared to those fed 90% (1881 g) and 100% AAs (1886 g) (*p* < 0.01). This trend persisted through day 41, with the 70% AA group weighing 2198 g, significantly lower than the 90% AA (2447 g) and 100% AA (2482 g) groups (*p* < 0.01). In terms of DBWG, broilers fed 70% AA exhibited the lowest DBWG across all AA levels from day 0 to 10 (19.75 g/day) and from day 10 to 24 (47.02 g/day) (*p* < 0.01 for both periods). From day 24 to 34, the 70% AA group also showed significantly lower DBWG (73.51 g/day) compared to broilers fed 90% AAs (82.60 g/day) (*p* = 0.02).

#### 3.1.2. Daily Feed Intake

Broilers fed 100% AAs and 84% AME exhibited a higher DFI (147.8 g/day) compared to those fed 70% AAs and 100% AME (112.5 g/day) during days 24–34 (*p* = 0.03, Table 6).

Additionally, broilers fed 100% AAs with 84% AME consistently consumed more feed than those fed 100% AAs with 92% AME during days 41–48, 48–55, and 55–62, with significant differences in DFI across these periods (*p* = 0.01, 0.01, and 0.05). The DFI of Cobb 700 broilers fed 84% AME was significantly higher than that of broilers fed 100% AME during days 10–24 and 34–41, with DFI values of 92.33 g/day and 179.1 g/day for the 84% AME group compared to 84.44 g/day and 162.5 g/day for the 100% AME group, respectively (*p* = 0.01 for both periods).

#### 3.1.3. Feed Conversion Ratio

From day 10 to 24, Cobb 700 broilers fed 70% and 80% AAs exhibited a higher FCR (1.82 and 1.70, respectively) compared to broilers fed 90% and 100% AAs (1.60 and 1.56, respectively) (*p* < 0.01, Table 7). During the same period, broilers fed 84% AME showed an increased FCR (1.74) compared to broilers fed 92% (1.64) and 100% AME (1.63) (*p* < 0.01). In addition, broilers fed 84% AME also exhibited a higher FCR than those fed 100% AME during both days 34–41 and day 41–48, with values of 2.23 and 2.44 for the 84% AME group compared to 2.00 and 2.14 for the 100% AME group (*p* = 0.01 and *p* < 0.01, respectively). When AME was set at 100%, Cobb 700 broilers fed 70% AA exhibited the highest FCR among all AA levels during days 0–10 (1.36, *p* = 0.04), further highlighting the negative impact of reduced AA levels on feed efficiency at early growth stages.

#### 3.1.4. Mortality

The mortality was not affected by the reduction in dietary AAs. However, Cobb 700 broilers fed 84% AME exhibited a higher percentage of mortality than broilers fed 100% AME during d 0–62 (*p* = 0.02, Table 8).

#### 3.1.5. Economic Analysis

Cobb 700 broilers fed 70% AAs exhibited a lower gross margin return (GMR)/bird than broilers fed 90% and 100% AAs at 41 d of age (*p* < 0.01, Table 13). On d 55, both broilers fed 70% and 80% AAs exhibited a lower feed cost/BW and a higher GMR/BW than broilers fed 100% AA (*p* < 0.01 and <0.01). Cobb 700 broilers fed 84% and 92% AME exhibited a lower feed cost/BW (*p* < 0.01) and a higher GMR/BW (*p* < 0.01) than broilers fed 100% AME at the age of d 41. Specifically, broilers fed 84% AME had a feed cost of USD 0.43/kg and a GMR/BW of USD 1.31 at day 41, whereas those on 100% AME showed a higher feed cost of USD 0.45/kg and a lower GMR/BW of USD 1.28.

### 3.2. Trial 2 of Ross 708 Broilers

#### 3.2.1. Body Weight and Daily Body Weight Gain

By day 64, Ross 708 broilers on the highest AA AME (AA 100%, AME 100%) diets achieved the greatest BW, reaching up to 3892 g, while the lowest-performing group (AA 70%, AME 100%) only reached a BW of 3092 g (*p* < 0.01, Table 9). Ross broilers with 70% dietary AAs exhibited a lower BW than broilers with 90% AAs at the age of d 64 (*p* < 0.01, Table 9). When dietary AA was at 70%, broilers fed 84% and 92% AME exhibited a higher BW than broilers with 100% AME before 55 d of age (all *p* < 0.01).

DBWG followed similar trends. Broilers in the AA 100%, AME 92% treatment exhibited the highest average daily gains during most intervals, with the highest BWG observed during the d 10–24 period at 56.6 g/day. In contrast, broilers fed 70% AAs with 100% AME had significantly lower gains during this same period (29.2 g/day, *p* = 0.01).

#### 3.2.2. Daily Feed Intake

During the initial phase (d 0–10), broilers on the 80%, 90%, and 100% AA diets exhibited significantly higher daily feed intake (25.44, 24.93, and 25.27, respectively, Table 10) compared to those on the 70% AA diet (22.65 g, *p* = 0.01). This trend continued throughout the feeding period, where higher AA and AME levels resulted in increased feed intake, particularly during the intermediate periods (d 24–34 and d 34–41). Broilers on the 70% AA and 84% AME treatment consumed the most feed by day 64, reaching 133.7 g/day (*p* = 0.01). In contrast, the lowest feed intake was observed in broilers fed the AA70, AME100 diet, with an average of 96.1 g/day by day 64.

#### 3.2.3. Feed Conversion Ratio

Ross 708 broilers fed 70% AAs exhibited the highest FCR (1.80) among all levels of AAs during d 10–24 (*p* < 0.01, Table 11). This trend continued through various phases, with higher AA and AME levels consistently associated with more efficient feed conversion. Broilers fed 84% AME showed a higher FCR (1.97) than broilers fed 100% AME during d 41–48 (*p* = 0.01). When dietary AA was at 90%, broilers fed 84% AME exhibited a higher FCR than broilers fed 92% and 100% AME during d 0–10 (*p* = 0.03).

#### 3.2.4. Mortality

The mortalities of Ross 708 broilers were not affected by the dietary treatments in all experimental periods (all *p* > 0.05, Table 12).

#### 3.2.5. Economic Analysis

Ross 708 broilers fed 70% and 80% AAs exhibited a lower feed cost/BW and a higher GMR/BW than broilers fed 100% AA at the age of d 41 (*p* = 0.01 and 0.01) and d 55 (*p* < 0.01 and <0.01, Table 13). On day 41, feed costs were USD 0.415 and USD 0.417, compared to USD 0.442 for 100% AA, and GMR/BW was 1.32 versus 1.29. This trend continued on day 55. In addition, broilers fed 84% and 92% AME exhibited a lower feed cost/BW and a higher GMR/BW than broilers fed 100% AME at the age of d 41 (*p* < 0.01 and <0.01) and 55 (*p* < 0.01 and <0.01). Broilers fed 70% AAs with 100% AME exhibited the lowest GMR/bird among all treatments at the age of d 41 (*p* = 0.01) and a lower GMR/bird than five other treatments at the age of d 55 (*p* < 0.01).

## 4. Discussion

The objective of the current study was to investigate the effects of dietary digestible AAs (TSAA, Lys, and Thr) and AME reduction on the growth performance and economic return of Cobb 700 and Ross 708 broilers.

### 4.1. Interaction Effects of AAs and AME

One of the key findings of this study was that the interaction between AAs and AME had a significant impact on broiler performance. For instance, Ross 708 broilers fed a diet with 70% AAs and 100% AME exhibited the lowest BW and feed intake (FI) between days 0 and 48. The decreased BW and FI of broilers were mainly due to an imbalanced ratio of energy to crude protein in this treatment, which had the highest ratio of energy to crude protein among all treatments in the current study. The imbalanced dietary ratio of energy to protein may change the availability of energy and amino acids, which are both needed in balanced quantities for the growth of chickens [15]. A previous study similarly showed that broilers fed a diet with a high ratio of AME to crude protein (3155 kcal/kg to 19.96%) exhibited a lower BWG than broilers fed a control diet with a normal ratio (2775 kcal/kg 20.83%) from 28 to 56 d of age [16]. In addition, this study also found that broilers fed a low-AME and -crude protein diet depressed BWG from 0 to 56 d of age as compared with broilers of a control diet. These results suggest that both nutritional density and ratio are essential factors influencing growth performance [17]. Therefore, the dietary nutritional reduction should be within a certain level and avoid unbalance between AAs and energy.

### 4.2. Effects of Dietary AA Reduction

In the current study, 10% and 20% dietary AA reductions did not affect the BW, BWG, or feed intake (FI) in Cobb 700 or Ross 708 broilers at 64 d of age. Zhang et al. [7] similarly found that three different broiler strains fed a 20% amino acid-reduced diet exhibited comparable body weight (BW) and feed intake (FI) to those fed a control diet from 0 to 55 days of age. However, previous studies have shown that broilers would increase FI to compensate for dietary AA dilution. For example, Ross 708 broilers fed a low-density AA diet (0.75% TSAA and 0.85% Lys) increased FI as compared with those fed a medium- (0.80% TSAA and 0.95% Lys) and high-density AA diet (0.83% TSAA and 1.05% Lys) during d 36–47 [18]. Zhai et al. [19] also reported that Cobb 700 broilers increased FI when dietary digestible AA density was decreased by 6% from 28 to 54 d of age. The inconsistent results between the current and previous studies are likely due to the different times of dietary AA reduction.

In contrast, Ross 708 broilers fed a 30% reduction in dietary AA had an increased FI from 55 to 64 d of age. However, the BW of Ross 708 broilers fed a 30% AA-reduced diet at 64 d of age was still lower than broilers fed a control diet without AA reduction. Broilers may not be able to maintain similar BW when they are continuously fed an AA-reduced diet. The BW and BWG of male birds linearly decreased when dietary AAs were continually reduced [20,21]. This decreased BW might be mainly due to the fact that a long period of feeding an AA-reduced diet may reduce the intake of dietary AAs, which are required for protein synthesis [4]. As a result, the protein deposition within the broilers’ body is decreased when dietary AAs are deficient [22]. The decreased BW and increased FI led to the increased FCR of Cobb 700 broilers fed 70% AA from 0 to 41 d in the current study. These results agreed with a previous study that Cobb 500 broilers fed a low-density AA diet had a higher FCR than broilers fed either a medium- or high-density AA diet [6]. Therefore, dietary AA reduction should be within a certain level and period to prevent adverse effects on the BW and feed utilization of broilers.

### 4.3. Effects of Dietary AME Reduction

In the current study, broilers continuously fed an AME-reduced diet exhibited compensatory growth. Broilers would exhibit compensatory growth when they were fed a nutrient-balanced diet after being fed a nutrient-reduced diet [18]. The BW of two strain broilers was not decreased by dietary AME reduction at 64 d of age. It has been suggested that broilers would increase FI to compensate for the dietary energy dilution [23]. As expected, the FI of broilers provided 84% AME was higher than that of broilers fed 100% AME in Cobb 700 before d 41 of age and in Ross 708 from 55 to 64 d of age in the present study. The increased FI could help broilers obtain a similar total caloric intake as a control group when broilers were fed an energy-reduced diet [5]. However, the increased FI led to a higher FCR in Ross 708 broilers fed 84% AME than in broilers fed 100% AME from 0 to 55 d of age. A previous study reported that the FCR was positively related to the FI [24]. Dozier III et al. [5] similarly observed that the FCR of Ross 308 broilers linearly increased when dietary AME was reduced from 3310 to 3175 kcal/kg from 30 to 47 d of age.

The growth performance was also affected by the interaction between AAs and energy. Ross 708 broilers fed 70% AAs with 100% AME exhibited the lowest BW and FI from 0 to 48 d. The decreased BW and FI of broilers were mainly due to an imbalanced ratio of energy to crude protein in this treatment, which had the highest ratio of energy to crude protein among all treatments in the current study. The imbalanced dietary ratio of energy to protein may change the availability of energy and amino acids, which are both needed in balanced quantities for the growth of chickens.

### 4.4. Economic Implications

The feed cost makes up around 60% of the total cost of production [25]. Dietary management is one of the methods to control feed costs and improve economic return. The feed cost/BW and gross margin return (GMR)/BW could reflect the price and marginal return per kilogram of BW, respectively. In the current study, the feed cost/BW was reduced, and the GMR/BW was increased in both strains of broilers fed either 20% and 30% dietary AA reduction or 8% and 16% dietary AME reduction. The GMR/bird could reflect the overall return per bird and depend on the BW of broilers. However, the GMR/bird was not improved by dietary reduction in AAs or AME in the current study. In contrast, the GMR/bird of Cobb 700 broilers fed a 30% AA-reduced diet was decreased at the age of d 41. In addition, the GMR/bird of Ross 708 broilers fed 70% AAs with 100% AME was decreased at 41 and 55 d, which may be due to the reduced BW of this treatment. Zhai et al. [21] similarly reported that the increased feed cost and feed cost/cut-up income were accompanied by decreased BW when dietary Met was reduced by 20%. Therefore, the dietary nutrient reduction should be within a certain level to prevent side effects on marginal return.

## 5. Conclusions

Reducing dietary AAs by 30% resulted in decreased BW, BWG, and gross return, while increasing FI and FCR. Reductions of 10% and 20% in dietary AAs did not negatively affect these parameters, indicating that moderate reductions can still support optimal growth and feed efficiency. Reducing AME by 16% increased FI and FCR, without significantly impacting the gross margin return. We recommend the following optimal dietary reductions for Cobb 700 and Ross 708 broilers: Starters (0–10 days): up to 20% AA and 8% AME reduction can be applied without affecting growth. Growers (11–24 days): 20% AA and 16% AME reduction maintain performance with compensatory growth. Finishers (25–39 days): 20% AA reduction is effective, but AME reduction beyond 16% may increase FCR. Withdrawals (40–64 days): AA reduction beyond 20% (e.g., 30%) decreases BW and GMR, while AME reduction beyond 16% increases FCR without economic benefit. Maintaining a balanced AA and energy ratio is crucial to optimize growth performance and economic return in broiler production.

## Figures and Tables

**Table 1:** Feed ingredient and nutrient composition of 12 dietary treatments with factorial combinations of 4 levels of digestible amino acids and 3 levels of apparent metabolizable energy during the Starter (d 0–10) feeding phase.

Parameter	Treatment											
AA (%) ^1^	70	70	70	80	80	80	90	90	90	100	100	100
AME (%)	84	92	100	84	92	100	84	92	100	84	92	100
Yellow Corn %	56.96	67.91	71.06	57.32	64.14	63.94	52.05	60.38	56.74	48.28	55.10	49.55
Soybean Meal %	29.90	22.74	22.27	29.38	28.24	28.27	35.14	33.74	34.35	40.64	39.50	40.43
Soybean Oil %	0.00	0.50	2.21	0.00	0.50	3.35	0.50	0.50	4.49	0.50	1.01	5.64
DL-Methionine %	0.18	0.23	0.23	0.29	0.28	0.28	0.33	0.32	0.33	0.38	0.37	0.38
L-Lysine HCl %	0.03	0.23	0.24	0.21	0.23	0.23	0.20	0.22	0.21	0.19	0.21	0.20
L-Threonine %	0.00	0.09	0.09	0.09	0.10	0.10	0.10	0.10	0.10	0.11	0.11	0.11
Ronozyme %	0.02	0.02	0.02	0.02	0.02	0.02	0.02	0.02	0.02	0.02	0.02	0.02
Dicalcium Phosphate %	1.74	1.75	1.74	1.74	1.73	1.73	1.72	1.71	1.72	1.70	1.69	1.70
Limestone %	1.45	1.47	1.48	1.44	1.45	1.45	1.42	1.44	1.43	1.41	1.42	1.41
Salt %	0.40	0.33	0.33	0.33	0.33	0.33	0.33	0.32	0.33	0.33	0.32	0.33
Premix % ^2^	0.25	0.25	0.25	0.25	0.25	0.25	0.25	0.25	0.25	0.25	0.25	0.25
Choline Chloride %	0.06	0.09	0.09	0.06	0.06	0.06	0.03	0.03	0.03	0.00	0.00	0.00
Sand %	9.00	4.39	0.00	8.87	2.68	0.00	7.90	0.96	0.00	6.19	0.00	0.00
Feed Price (USD/kg)	0.24	0.24	0.26	0.25	0.26	0.28	0.26	0.27	0.29	0.27	0.28	0.31
Calculated Composition												
CP, %	16.48	16.46	16.49	18.77	18.79	18.79	21.10	21.11	21.11	23.42	23.44	23.43
Ca, %	0.96	0.96	0.96	0.96	0.96	0.96	0.96	0.96	0.96	0.96	0.96	0.96
Available *P* %	0.48	0.48	0.48	0.48	0.48	0.48	0.48	0.48	0.48	0.48	0.48	0.48
M.E. (kcal/kg)	2550	2792	3035	2549	2792	3035	2549	2792	3035	2549	2792	3035
Digestible Met %	0.44	0.46	0.46	0.54	0.53	0.53	0.61	0.60	0.61	0.68	0.68	0.68
Digestible TSAAs %	0.66	0.66	0.66	0.76	0.76	0.76	0.85	0.85	0.85	0.95	0.95	0.95
Digestible Lys %	0.90	0.90	0.90	1.02	1.02	1.02	1.15	1.15	1.15	1.28	1.28	1.28
Digestible Thr %	0.60	0.60	0.60	0.69	0.69	0.69	0.77	0.77	0.77	0.86	0.86	0.86
Digestible Try %	0.22	0.18	0.18	0.21	0.21	0.21	0.24	0.24	0.24	0.27	0.27	0.27
Digestible Leu %	1.47	1.33	1.34	1.45	1.48	1.47	1.60	1.62	1.61	1.74	1.77	1.75
Digestible Val %	0.78	0.67	0.67	0.77	0.77	0.77	0.86	0.86	0.86	0.96	0.96	0.96
Digestible Arg %	1.15	0.96	0.96	1.14	1.13	1.13	1.30	1.29	1.29	1.47	1.45	1.46
Choline (ppm)	789	789	789	789	789	789	789	789	789	789	788	789
Chloride %	0.27	0.27	0.27	0.26	0.26	0.26	0.25	0.25	0.25	0.24	0.24	0.24
Sodium %	0.19	0.16	0.16	0.16	0.16	0.16	0.16	0.16	0.16	0.16	0.16	0.16
ME/CP (kcal/kg/%)	136.3	169.6	184.1	135.8	148.6	161.5	120.8	132.3	143.8	108.9	119.1	129.5

^1^ Amino acids in the 100% diet were at the higher recommended levels of digestible amino acids (lysine, TSAAs, and threonine). ^2^ Premix did not contain riboflavin and provided the following per kilogram of finished diet: retinyl acetate, 2.654 μg; cholecalciferol, 110 μg; DL-α-tocopherol acetate, 9.9 mg; menadione, 0.9 mg; vitamin B12, 0.01 mg; folic acid, 0.6 μg; choline, 379 mg; D-pantothenic acid, 8.8 mg; niacin, 33 mg; thiamine, 1.0 mg; D-biotin, 0.1 mg; pyridoxine, 0.9 mg; ethoxyquin, 28 mg; manganese, 55 mg; zinc, 50 mg; iron, 28 mg; copper, 4 mg; iodine, 0.5 mg; selenium, 0.1 mg.

**Table 2:** Feed ingredient and nutrient composition of 12 dietary treatments with factorial combinations of 4 levels of digestible amino acids and 3 levels of apparent metabolizable energy during the Grower (d10–24) feeding phase.

Parameter	Treatment											
AA (%) ^1^	70	70	70	80	80	80	90	90	90	100	100	100
AME (%)	84	92	100	84	92	100	84	92	100	84	92	100
Yellow Corn %	62.18	72.39	74.46	62.24	69.27	68.45	57.33	65.86	61.93	53.92	61.10	55.41
Soybean Meal %	25.00	19.17	18.82	24.90	23.73	23.86	30.14	28.71	29.37	35.13	33.93	34.88
Soybean Oil %	0.00	0.50	2.65	0.00	0.50	3.62	0.50	0.50	4.66	0.50	0.94	5.69
DL-Methionine %	0.17	0.20	0.20	0.26	0.25	0.25	0.30	0.29	0.30	0.34	0.34	0.34
L-Lysine HCl %	0.06	0.22	0.23	0.21	0.23	0.22	0.20	0.22	0.21	0.19	0.21	0.19
L-Threonine %	0.00	0.07	0.07	0.08	0.08	0.08	0.09	0.09	0.09	0.09	0.09	0.09
Ronozyme %	0.02	0.02	0.02	0.02	0.02	0.02	0.02	0.02	0.02	0.02	0.02	0.02
Dicalcium Phosphate %	1.51	1.51	1.51	1.51	1.50	1.50	1.49	1.48	1.48	1.48	1.46	1.47
Limestone %	1.36	1.38	1.38	1.35	1.36	1.36	1.33	1.35	1.34	1.32	1.33	1.32
Salt %	0.40	0.33	0.33	0.33	0.33	0.33	0.33	0.33	0.33	0.33	0.32	0.33
Premix % ^2^	0.25	0.25	0.25	0.25	0.25	0.25	0.25	0.25	0.25	0.25	0.25	0.25
Choline Chloride %	0.05	0.08	0.08	0.06	0.06	0.06	0.03	0.03	0.03	0.00	0.00	0.00
Sand %	9.00	3.88	0.00	8.80	2.43	0.00	7.99	0.88	0.00	6.44	0.00	0.00
Feed Price (USD/kg)	0.23	0.24	0.25	0.24	0.25	0.27	0.25	0.26	0.28	0.26	0.27	0.30
Calculated Composition												
CP %	15.09	15.09	15.09	17.02	17.03	17.03	19.13	19.14	19.13	21.23	21.25	21.24
Ca %	0.87	0.87	0.87	0.87	0.87	0.87	0.87	0.87	0.87	0.87	0.87	0.87
Available *P* %	0.44	0.44	0.43	0.43	0.43	0.44	0.44	0.44	0.44	0.43	0.43	0.43
M.E. (kcal/kg)	2611	2859	3108	2611	2859	3108	2611	2859	3108	2611	2859	3108
Digestible Met %	0.40	0.42	0.42	0.49	0.48	0.49	0.55	0.55	0.55	0.62	0.62	0.62
Digestible TSAA %	0.61	0.61	0.61	0.70	0.70	0.70	0.78	0.78	0.78	0.87	0.87	0.87
Digestible Lys %	0.80	0.80	0.81	0.92	0.92	0.92	1.03	1.04	1.03	1.15	1.15	1.15
Digestible Thr %	0.54	0.54	0.54	0.62	0.62	0.62	0.69	0.69	0.69	0.77	0.77	0.77
Digestible Try %	0.19	0.16	0.16	0.19	0.19	0.19	0.22	0.22	0.22	0.25	0.24	0.24
Digestible Leu %	1.35	1.25	1.26	1.35	1.37	1.37	1.48	1.51	1.49	1.61	1.63	1.62
Digestible Val %	0.70	0.62	0.62	0.70	0.70	0.70	0.78	0.78	0.78	0.87	0.87	0.87
Digestible Arg %	1.02	0.86	0.86	1.01	1.00	1.00	1.16	1.15	1.15	1.31	1.31	1.31
Choline (ppm)	729	729	729	729	729	729	729	729	729	730	729	730
Chloride %	0.27	0.27	0.27	0.26	0.26	0.26	0.25	0.25	0.25	0.24	0.24	0.24
Sodium %	0.19	0.16	0.16	0.16	0.16	0.16	0.16	0.16	0.16	0.16	0.16	0.16
ME/CP (kcal/kg/%)	155.3	189.5	206.0	153.4	167.9	182.5	136.5	149.4	162.5	123.0	134.5	146.3

^1^ Amino acids in the 100% diet were at the higher recommended levels of digestible amino acids (lysine, TSAAs, and threonine). ^2^ Premix did not contain riboflavin and provided the following per kilogram of finished diet: retinyl acetate, 2.654 μg; cholecalciferol, 110 μg; DL-α-tocopherol acetate, 9.9 mg; menadione, 0.9 mg; vitamin B12, 0.01 mg; folic acid, 0.6 μg; choline, 379 mg; D-pantothenic acid, 8.8 mg; niacin, 33 mg; thiamine, 1.0 mg; D-biotin, 0.1 mg; pyridoxine, 0.9 mg; ethoxyquin, 28 mg; manganese, 55 mg; zinc, 50 mg; iron, 28 mg; copper, 4 mg; iodine, 0.5 mg; selenium, 0.1 mg.

**Table 3:** Feed ingredient and nutrient composition of 12 dietary treatments with factorial combinations of 4 levels of digestible amino acids and 3 levels of apparent metabolizable energy during the Finisher (d 24–41) feeding phase.

Parameter	Treatment											
AA (%) ^1^	70	70	70	80	80	80	90	90	90	100	100	100
AME (%)	84	92	100	84	92	100	84	92	100	84	92	100
Yellow Corn %	67.99	75.30	73.54	64.37	72.78	68.94	61.57	69.16	63.72	58.76	63.94	58.50
Soybean Meal %	19.96	18.67	18.98	23.28	22.36	22.78	27.38	26.55	27.15	31.48	30.92	31.51
Soybean Oil %	0.00	0.50	4.01	0.50	0.50	4.77	0.50	0.78	5.61	0.50	1.63	6.45
DL-Methionine %	0.16	0.15	0.15	0.21	0.20	0.20	0.25	0.24	0.25	0.30	0.29	0.30
L-Lysine HCl %	0.09	0.11	0.11	0.12	0.13	0.13	0.13	0.13	0.13	0.13	0.14	0.13
L-Threonine %	0.00	0.01	0.01	0.03	0.02	0.03	0.04	0.03	0.04	0.05	0.05	0.05
Ronozyme %	0.02	0.02	0.02	0.02	0.02	0.02	0.02	0.02	0.02	0.02	0.02	0.02
Dicalcium Phosphate %	1.28	1.26	1.27	1.27	1.25	1.26	1.25	1.24	1.25	1.24	1.23	1.24
Limestone %	1.26	1.27	1.27	1.25	1.26	1.25	1.23	1.25	1.24	1.22	1.23	1.22
Salt %	0.33	0.33	0.33	0.33	0.33	0.33	0.33	0.33	0.33	0.33	0.33	0.33
Premix % ^2^	0.25	0.25	0.25	0.25	0.25	0.25	0.25	0.25	0.25	0.25	0.25	0.25
Choline Chloride %	0.06	0.06	0.06	0.05	0.04	0.04	0.02	0.02	0.02	0.00	0.00	0.00
Sand %	8.60	2.06	0.00	8.32	0.86	0.00	7.02	0.00	0.00	5.72	0.00	0.00
Feed Price (USD/kg)	0.22	0.23	0.25	0.23	0.24	0.26	0.24	0.25	0.28	0.25	0.26	0.29
Calculated Composition												
CP %	14.92	14.90	14.91	16.28	16.50	16.40	18.03	18.23	18.09	19.78	19.92	19.77
Ca %	0.78	0.78	0.78	0.78	0.78	0.78	0.78	0.78	0.78	0.78	0.78	0.78
Available *P* %	0.39	0.39	0.39	0.39	0.39	0.39	0.39	0.39	0.39	0.39	0.39	0.39
M.E. (kcal/kg)	2689	2944	3200	2688	2944	3200	2688	2944	3200	2688	2944	3200
Digestible Met %	0.37	0.37	0.37	0.44	0.43	0.43	0.50	0.49	0.50	0.56	0.56	0.56
Digestible TSAA %	0.56	0.56	0.56	0.64	0.64	0.64	0.72	0.72	0.72	0.80	0.80	0.80
Digestible Lys %	0.71	0.71	0.71	0.82	0.82	0.82	0.92	0.92	0.92	1.02	1.02	1.02
Digestible Thr %	0.48	0.48	0.48	0.54	0.54	0.54	0.61	0.61	0.61	0.68	0.68	0.68
Digestible Try %	0.17	0.16	0.16	0.18	0.18	0.18	0.20	0.21	0.20	0.23	0.23	0.23
Digestible Leu %	1.24	1.26	1.25	1.31	1.36	1.34	1.42	1.46	1.44	1.53	1.56	1.53
Digestible Val %	0.62	0.62	0.62	0.67	0.68	0.68	0.74	0.75	0.75	0.81	0.82	0.81
Digestible Arg %	0.87	0.86	0.86	0.97	0.97	0.97	1.09	1.09	1.09	1.21	1.21	1.21
Choline (ppm)	693	693	693	693	693	693	693	693	693	693	696	693
Chloride %	0.24	0.24	0.24	0.24	0.24	0.24	0.23	0.23	0.23	0.23	0.23	0.23
Sodium %	0.16	0.16	0.16	0.16	0.16	0.16	0.16	0.16	0.16	0.16	0.16	0.16
ME/CP (kcal/kg/%)	180.2	197.6	214.6	165.1	178.4	195.1	149.1	161.5	176.9	135.9	147.8	161.9

^1^ Amino acids in the 100% diet were at the higher recommended levels of digestible amino acids (lysine, TSAAs, and threonine). ^2^ Premix did not contain riboflavin and provided the following per kilogram of finished diet: retinyl acetate, 2.654 μg; cholecalciferol, 110 μg; DL-α-tocopherol acetate, 9.9 mg; menadione, 0.9 mg; vitamin B12, 0.01 mg; folic acid, 0.6 μg; choline, 379 mg; D-pantothenic acid, 8.8 mg; niacin, 33 mg; thiamine, 1.0 mg; D-biotin, 0.1 mg; pyridoxine, 0.9 mg; ethoxyquin, 28 mg; manganese, 55 mg; zinc, 50 mg; iron, 28 mg; copper, 4 mg; iodine, 0.5 mg; selenium, 0.1 mg.

**Table 4:** Feed ingredient and nutrient composition of 12 dietary treatments with factorial combinations of 4 levels of digestible amino acids and 3 levels of apparent metabolizable energy during the Withdrawal (d 41–64) feeding phase.

Parameter	Treatment											
AA (%) ^1^	70	70	70	80	80	80	90	90	90	100	100	100
AME (%)	84	92	100	84	92	100	84	92	100	84	92	100
Yellow Corn	68.69	77.60	73.34	67.61	76.08	70.69	64.94	71.21	65.74	62.28	66.25	60.78
Soybean Meal	19.84	18.27	19.02	21.42	20.49	21.08	25.32	24.63	25.23	29.22	28.78	29.38
Soybean Oil	0.00	0.00	4.37	0.00	0.00	4.83	0.00	0.77	5.63	0.00	1.57	6.43
DL-Methionine	0.12	0.11	0.12	0.18	0.17	0.18	0.22	0.21	0.22	0.26	0.26	0.27
L-Lysine HCl	0.04	0.07	0.05	0.11	0.12	0.12	0.12	0.12	0.12	0.12	0.12	0.12
L-Threonine	0.00	0.00	0.00	0.02	0.01	0.02	0.03	0.02	0.03	0.04	0.03	0.04
Ronozyme	0.02	0.02	0.02	0.02	0.02	0.02	0.02	0.02	0.02	0.02	0.02	0.02
Dicalcium Phosphate	1.22	1.21	1.21	1.21	1.20	1.21	1.20	1.19	1.20	1.19	1.18	1.19
Limestone	1.24	1.25	1.24	1.23	1.24	1.24	1.22	1.23	1.22	1.21	1.21	1.20
Salt	0.33	0.33	0.33	0.33	0.33	0.33	0.33	0.33	0.33	0.33	0.33	0.33
Premix ^2^	0.25	0.25	0.25	0.25	0.25	0.25	0.25	0.25	0.25	0.25	0.25	0.25
Choline Chloride	0.05	0.05	0.05	0.04	0.04	0.04	0.02	0.02	0.02	0.00	0.00	0.00
Sand	8.20	0.84	0.000.00	7.57	0.05	0.00.	6.33	0.00	0.00	5.10	0.00	0.00
Feed Price (USD/kg)	0.22	0.23	0.25	0.23	0.23	0.26	0.23	0.25	0.27	0.24	0.26	0.29
Calculated Composition												
CP, %	14.84	14.83	14.83	15.62	15.85	15.70	17.28	17.45	17.31	18.95	19.05	18.91
Ca, %	0.76	0.76	0.76	0.76	0.76	0.76	0.76	0.76	0.76	0.76	0.76	0.76
Available *P*, %	0.38	0.38	0.38	0.38	0.38	0.38	0.38	0.38	0.38	0.38	0.38	0.38
M.E. (kcal/kg)	2709	2967	3225	2709	2967	3225	2709	2967	3225	2709	2967	3225
Digestible Met, %	0.33	0.33	0.33	0.40	0.40	0.40	0.46	0.46	0.46	0.52	0.52	0.52
Digestible TSAA, %	0.53	0.53	0.53	0.60	0.60	0.60	0.67	0.67	0.67	0.75	0.75	0.75
Digestible Lys, %	0.67	0.67	0.67	0.77	0.77	0.77	0.86	0.86	0.86	0.96	0.96	0.96
Digestible Thr, %	0.47	0.47	0.47	0.51	0.51	0.51	0.58	0.58	0.58	0.64	0.64	0.64
Digestible Try	0.17	0.16	0.16	0.17	0.17	0.17	0.19	0.20	0.19	0.22	0.22	0.22
Digestible Leu	1.24	1.26	1.25	1.28	1.32	1.30	1.39	1.42	1.39	1.49	1.51	1.48
Digestible Val	0.62	0.62	0.62	0.65	0.65	0.65	0.71	0.72	0.71	0.78	0.79	0.78
Digestible Arg	0.87	0.85	0.86	0.92	0.92	0.92	1.04	1.03	1.03	1.15	1.15	1.15
Choline (ppm)	670	670	670	670	670	670	670	670	670	671	674	671
Chloride, %	0.22	0.23	0.23	0.24	0.23	0.24	0.23	0.23	0.23	0.22	0.22	0.22
Sodium, %	0.16	0.16	0.16	0.16	0.16	0.16	0.16	0.16	0.16	0.16	0.16	0.16
ME/CP (kcal/kg/%)	182.5	200.1	217.5	173.4	187.2	205.4	156.8	170.0	186.3	143.0	155.7	170.5

^1^ Amino acids in the 100% diet were at the higher recommended levels of digestible amino acids (lysine, TSAAs, and threonine). ^2^ Premix did not contain riboflavin and provided the following per kilogram of finished diet: retinyl acetate, 2.654 μg; cholecalciferol, 110 μg; DL-α-tocopherol acetate, 9.9 mg; menadione, 0.9 mg; vitamin B12, 0.01 mg; folic acid, 0.6 μg; choline, 379 mg; D-pantothenic acid, 8.8 mg; niacin, 33 mg; thiamine, 1.0 mg; D-biotin, 0.1 mg; pyridoxine, 0.9 mg; ethoxyquin, 28 mg; manganese, 55 mg; zinc, 50 mg; iron, 28 mg; copper, 4 mg; iodine, 0.5 mg; selenium, 0.1 mg.

**Table 5:** Body weight and daily body weight gain of Cobb 700 broilers fed 12 diets from 0 to 62 d of age.

Treatment		BW (g)								Daily BW Gain (g/day)						
AA (%)	AME (%)	D0	D10	D24	D34	D41	D48	D55	D62 ^1^	D0–10	D10–24	D24–34	D34–41	D41–48	D48–55 ^1^	D55–62 ^1^
70		43.29	240.8 ^b^	899 ^b^	1634 ^b^	2198 ^b^	2750	3165	3546	19.75 ^b^	47.02 ^c^	73.51 ^b^	80.49	78.85	61.38	54.8
80		43.41	256.7 ^a^	993 ^a^	1757 ^ab^	2335 ^ab^	2905	3302	3593	21.33 ^a^	52.57 ^b^	76.48 ^ab^	82.55	80.50	58.31	44.1
90		43.17	262.5 ^a^	1055 ^a^	1881 ^a^	2447 ^a^	2983	3342	3716	21.93 ^a^	56.60 ^ab^	82.60 ^a^	80.81	78.40	55.78	45.4
100		43.02	259.6 ^a^	1071 ^a^	1886 ^a^	2482 ^a^	3036	3422	3799	21.66 ^a^	57.99 ^a^	81.47 ^ab^	85.17	79.10	55.18	47.0
SEM ^2^		0.22	3.77	21.40	37.80	47.70	61.80	76.20	77.40	0.38	1.31	2.23	2.85	3.29	3.52	4.27
	84	43.06	254.2	1001	1801	2375	2897	3300	3717	21.11	53.37	79.91	82.11	77.11	57.89	48.5
	92	43.37	256.3	1023	1817	2396	2938	3313	3535	21.29	54.79	79.32	82.76	76.25	56.59	44.0
	100	43.24	254.2	989	1752	2325	2920	3310	3739	21.10	52.46	76.32	81.89	84.28	58.50	51.0
	SEM	0.19	3.26	18.50	32.70	41.30	53.50	65.90	67.20	0.33	1.13	1.93	2.47	2.85	3.05	3.83
70	84						2831 ^ab^	3304 ^ab^	3676 ^a^						67.58 ^a^	47.5 ^a^
70	92						2787 ^ab^	3213 ^ab^	3491 ^a^						60.87 ^a^	54.8 ^a^
70	100						2631 ^b^	2977 ^b^	3472 ^a^						55.68 ^a^	62.1 ^a^
80	84						2836 ^ab^	3221 ^ab^	3675 ^a^						55.05 ^a^	51.1 ^a^
80	92						2886 ^ab^	3255 ^ab^	3531 ^a^						52.76 ^a^	39.4 ^a^
80	100						2995 ^ab^	3431 ^ab^	3573 ^a^						67.12 ^a^	41.8 ^a^
90	84						2705 ^ab^	3011 ^ab^	3406 ^a^						44.93 ^a^	32.0 ^a^
90	92						3166 ^a^	3542 ^ab^	3780 ^a^						65.70 ^a^	55.1 ^a^
90	100						3078 ^ab^	3475 ^ab^	3962 ^a^						56.70 ^a^	49.2 ^a^
100	84						3217 ^a^	3665 ^a^	4113 ^a^						64.01 ^a^	63.3 ^a^
100	92						2914 ^ab^	3243 ^ab^	3337 ^a^						47.05 ^a^	26.9 ^a^
100	100						2977 ^ab^	3358 ^ab^	3948 ^a^						54.49 ^a^	50.9 ^a^
SEM							107.10	131.90	134.30						6.09	7.67
*p*-value																
AA		0.63	<0.01	<0.01	<0.01	<0.01	0.01	0.12	0.10	<0.01	<0.01	0.02	0.62	0.96	0.61	0.37
AME		0.68	0.87	0.31	0.35	0.47	1.00	1.00	0.06	0.89	0.27	0.39	0.98	0.11	0.90	0.38
AA × AME		0.42	0.80	0.38	0.21	0.08	0.02	0.01	0.01	0.83	0.33	0.25	0.16	0.24	0.04	0.04

^a–c^ Means in a column not sharing a common superscript were different (*p* < 0.05). ^1^ Tukey’s test was not able to separate treatment means of BW at d 62, BWG during d 48–55 and d 55–62. ^2^ SEM = standard error of mean.

**Table 6:** Daily feed intake of Cobb 700 broilers fed 12 diets from 0 to 62 d of age.

Treatment		Daily Feed Intake (g/day)												
AA (%)	AME (%)	D0–10	D10–24	D24–34	D34–41	D41–48	D48–55	D55–62	D0–24	D0–34	D0–41	D0–48	D0–55	D0–62
70		26.51	85.04	129.7	174.4	179.9	168.9	175.0	60.65	80.95	96.91	109.0	116.5	123.3
80		27.58	89.16	137.7	169.5	179.5	159.1	162.4	63.50	85.33	99.71	111.7	117.9	122.9
90		26.71	90.27	136.5	168.1	172.7	156.0	161.3	63.79	85.17	99.33	109.3	115.6	122.1
100		26.10	90.22	134.7	170.0	178.5	161.1	170.0	63.50	84.43	99.04	110.6	117.1	122.1
SEM ^1^		0.54	1.93	3.42	4.07	4.95	5.78	6.28	1.31	1.71	1.86	2.17	2.43	2.65
	84	27.33	92.33 ^a^	139.5	179.1 ^a^	183.4	169.6	181.1	65.25 ^a^	87.08 ^a^	102.79 ^a^	113.9	121.0	128.3
	92	26.72	89.25 ^ab^	136.9	170.0 ^ab^	174.3	158.4	158.4	63.19 ^ab^	84.86 ^ab^	99.39 ^ab^	110.4	116.8	120.9
	100	26.13	84.44 ^b^	127.6	162.5 ^b^	175.3	155.8	162.0	60.14 ^b^	79.97 ^b^	94.06 ^b^	106.2	112.6	118.6
	SEM	0.47	1.67	2.96	3.52	4.28	5.00	5.44	1.14	1.49	1.61	1.88	2.11	2.29
70	84			142.0 ^a^		195.4 ^ab^	186.8 ^a^	186.1 ^ab^				117.9 ^a^	126.7 ^ab^	133.4 ^a^
70	92			134.5 ^ab^		177.7 ^ab^	171.8 ^ab^	164.6 ^ab^				109.8 ^ab^	117.6 ^abc^	122.9 ^ab^
70	100			112.5 ^b^		166.7 ^ab^	148.1 ^ab^	174.3 ^ab^				99.4 ^b^	105.3 ^c^	113.5 ^ab^
80	84			136.1 ^ab^		175.9 ^ab^	158.5 ^ab^	178.4 ^ab^				111.9 ^ab^	117.8 ^abc^	123.2 ^ab^
80	92			137.7 ^ab^		176.9 ^ab^	152.7 ^ab^	160.0 ^ab^				111.2 ^ab^	116.5 ^abc^	121.4 ^ab^
80	100			139.4 ^ab^		185.6 ^ab^	166.2 ^ab^	148.9 ^ab^				112.0 ^ab^	119.5 ^abc^	124.1 ^ab^
90	84			132.1 ^ab^		160.3 ^ab^	145.2 ^ab^	153.0 ^ab^				104.0 ^ab^	109.3 ^bc^	118.5 ^ab^
90	92			140.2 ^ab^		184.7 ^ab^	171.0 ^ab^	171.4 ^ab^				115.2 ^ab^	123.3 ^abc^	128.4 ^ab^
90	100			137.2 ^ab^		173.0 ^ab^	151.9 ^ab^	159.5 ^ab^				108.8 ^ab^	114.3 ^abc^	119.4 ^ab^
100	84			147.8 ^a^		201.9 ^a^	187.8 ^a^	206.9 ^a^				121.8 ^a^	130.2 ^a^	138.2 ^a^
100	92			135.0 ^ab^		157.9 ^b^	138.3 ^b^	137.7 ^b^				105.6 ^ab^	109.7 ^bc^	110.7 ^b^
100	100			121.2 ^ab^		175.8 ^ab^	157.1 ^ab^	165.4 ^ab^				104.5 ^ab^	111.2 ^abc^	117.3 ^ab^
SEM				5.92		8.57	10.00	10.88				3.76	4.21	4.59
*p*-value														
AA		0.26	0.23	0.38	0.79	0.85	0.53	0.38	0.25	0.19	0.65	0.84	0.96	0.92
AME		0.17	0.01	0.02	0.01	0.14	0.13	0.03	0.01	<0.01	<0.01	0.02	0.03	0.03
AA × AME		0.79	0.44	0.03	0.05	0.01	0.01	0.05	0.65	0.13	0.07	0.01	<0.01	0.01

^a–c^ Means in a column not sharing a common superscript were different (*p* < 0.05). ^1^ SEM = standard error of mean.

**Table 7:** Feed conversion ratio of Cobb 700 broilers fed 12 diets from 0 to 62 d of age.

Treatment		FCR												
AA (%)	AME (%)	D0–10	D10–24	D24–34 ^1^	D34–41	D41–48	D48–55	D55–62	D0–24	D0–34	D0–41	D0–48	D0–55	D0–62
70		1.34	1.82 ^a^	1.76 ^a^	2.18	2.34	2.90	2.91	1.71 ^a^	1.73 ^a^	1.84 ^a^	1.94 ^a^	2.06 ^a^	2.14 ^a^
80		1.29	1.70 ^b^	1.82 ^a^	2.10	2.30	2.84	3.06	1.61 ^b^	1.69 ^a^	1.79 ^a^	1.88 ^ab^	1.99 ^ab^	2.08 ^ab^
90		1.22	1.60 ^c^	1.67 ^a^	2.15	2.26	2.91	3.10	1.52 ^c^	1.59 ^b^	1.70 ^b^	1.80 ^bc^	1.92 ^b^	2.04 ^b^
100		1.21	1.56 ^c^	1.67 ^a^	2.03	2.31	3.09	3.19	1.49 ^c^	1.56 ^b^	1.67 ^b^	1.78 ^c^	1.91 ^b^	2.01 ^b^
SEM ^2^		0.01	0.02	0.04	0.06	0.06	0.12	0.13	0.02	0.02	0.02	0.02	0.02	0.02
	84	1.30	1.74 ^a^	1.77	2.23 ^a^	2.44 ^a^	3.07	3.35	1.64 ^a^	1.69 ^a^	1.82 ^a^	1.93 ^a^	2.06 ^a^	2.17 ^a^
	92	1.26	1.64 ^b^	1.74	2.11 ^ab^	2.33 ^a^	2.95	3.11	1.56 ^b^	1.64 ^ab^	1.74 ^b^	1.84 ^b^	1.96 ^b^	2.06 ^b^
	100	1.24	1.63 ^b^	1.68	2.00 ^b^	2.14 ^b^	2.79	2.74	1.54 ^b^	1.60 ^b^	1.70 ^b^	1.78 ^b^	1.90 ^b^	1.97 ^c^
	SEM	0.01	0.02	0.04	0.05	0.06	0.11	0.11	0.01	0.02	0.02	0.02	0.02	0.02
70	84	1.33 ^abc^						3.42 ^ab^						
70	92	1.34 ^ab^						2.73 ^b^						
70	100	1.36 ^a^						2.59 ^b^						
80	84	1.34 ^ab^						2.89 ^ab^						
80	92	1.30 ^abcd^						3.32 ^ab^						
80	100	1.23 ^bcde^						2.98 ^ab^						
90	84	1.27 ^abcde^						4.08 ^a^						
90	92	1.22 ^cde^						2.58 ^b^						
90	100	1.18 ^e^						2.64 ^b^						
100	84	1.25 ^bcde^						3.03 ^ab^						
100	92	1.18 ^e^						3.80 ^a^						
100	100	1.19 ^de^						2.74 ^b^						
SEM		0.02						0.22						
*p*-value														
AA		<0.01	<0.01	0.04	0.29	0.79	0.50	0.55	<0.01	<0.01	<0.01	<0.01	<0.01	<0.01
AME		<0.01	<0.01	0.15	0.01	<0.01	0.25	<0.01	<0.01	<0.01	<0.01	<0.01	<0.01	<0.01
AA × AME		0.04	0.30	0.41	0.86	0.99	0.38	<0.01	0.12	0.77	0.84	0.83	0.82	0.39

^a–e^ Means in a column not sharing a common superscript were different (*p* < 0.05). ^1^ Tukey’s test was not able to separate treatments means of FCR during d 24–34. ^2^ SEM = standard error of mean.

**Table 8:** Mortality (dead birds/total birds) of Cobb 700 broilers fed 12 diets from 0 to 62 d of age.

Treatment		Mortality (%)												
AA (%)	AME (%)	D0–10	D10–24	D24–34	D34–41	D41–48	D48–55	D55–62	D0–24	D0–34	D0–41	D0–48	D0–55	D0–62
70		2.78	1.85	0.00	0.00	0.00	0.00	0.46	4.63	4.63	4.63	4.63	4.63	5.09
80		2.31	1.39	0.46	0.00	0.46	0.93	0.93	3.70	4.17	4.17	4.63	5.56	6.48
90		3.70	1.39	0.93	0.46	0.46	1.39	0.46	5.09	6.02	6.48	6.94	8.33	8.80
100		0.46	3.24	0.93	0.46	0.00	1.39	1.39	3.70	4.63	5.09	5.09	6.48	7.87
SEM ^1^		1.08	1.11	0.51	0.33	0.33	0.71	0.67	1.65	1.67	1.64	1.62	1.70	1.85
	84	2.43	2.78	1.04	0.00	0.35	1.74	1.39	5.21	6.25	6.25	6.60	8.33	9.72 ^a^
	92	2.08	2.08	0.69	0.69	0.35	1.04	1.04	4.17	4.86	5.56	5.90	6.94	7.99 ^ab^
	100	2.43	1.04	0.00	0.00	0.00	0.00	0.00	3.47	3.47	3.47	3.47	3.47	3.47 ^b^
	SEM	0.94	0.96	0.44	0.29	0.29	0.61	0.58	1.43	1.45	1.42	1.41	1.47	1.60
*p*-value														
AA		0.14	0.57	0.48	0.63	0.52	0.48	0.51	0.88	0.82	0.71	0.62	0.40	0.55
AME		0.90	0.46	0.23	0.15	0.62	0.14	0.23	0.67	0.39	0.35	0.26	0.06	0.02
AA × AME		0.64	0.98	0.92	0.69	0.31	0.63	0.55	0.85	0.79	0.62	0.61	0.51	0.44

^a,b^ Means in a column not sharing a common superscript were different (*p* < 0.05). ^1^ SEM = standard error of mean.

**Table 9:** Body weight and daily body weight gain of Ross 708 broilers fed 12 diets from 0 to 64 d of age.

Treatment		BW (g)								Daily BW Gain (g/day)						
AA (%)	AME (%)	D0	D10	D24	D34	D41	D48	D55	D64	D0–10	D10–24	D24–34	D34–41	D41–48	D48–55 ^1^	D55–64
70		42.02	221.3	779	1426	1990	2566	3087	3453 ^b^	17.93	39.8	64.7	80.6	82.4	73.5	75.8 ^a^
80		41.83	246.0	946	1678	2256	2834	3299	3724 ^ab^	20.42	50.0	73.3	82.6	81.6	64.6	52.4 ^b^
90		41.98	248.2	966	1729	2288	2756	3131	3926 ^a^	20.62	51.3	76.3	79.8	77.2	59.1	50.6 ^b^
100		42.21	251.2	1008	1788	2392	2971	3361	3699 ^ab^	20.90	54.0	78.0	86.3	81.6	58.1	46.4 ^b^
SEM ^2^		0.27	4.34	25.30	47.40	63.30	79.20	84.90	80.40	0.42	1.55	2.44	3.21	3.14	3.68	4.14
	84	42.31	243.5	942	1693	2290	2829	3236	3833	20.12	49.9	75.1	85.3	82.8	65.6	56.4
	92	42.02	249.4	966	1735	2343	2917	3371	3687	20.74	51.2	76.9	86.8	82.1	63.6	59.4
	100	41.71	232.1	866	1538	2061	2600	3050	3582	19.04	45.3	67.1	74.8	77.3	62.2	53.0
	SEM	0.23	3.75	21.90	41.00	54.80	68.50	73.50	69.60	0.37	1.34	2.11	2.78	2.72	3.19	3.58
70	84		240.6 ^a^	861 ^a^	1558 ^a^	2166 ^a^	2813 ^ab^	3354 ^a^	3787	19.82 ^a^	44.3 ^a^	69.7 ^ab^	86.9 ^ab^	92.5 ^a^	77.2 ^a^	
70	92		234.1 ^a^	877 ^a^	1625 ^a^	2268 ^a^	2883 ^ab^	3392 ^a^	3480	19.21 ^a^	45.9 ^a^	74.8 ^a^	91.8 ^a^	88.0 ^ab^	72.7 ^a^	
70	100		189.3 ^b^	598 ^b^	1094 ^b^	1535 ^b^	2002 ^c^	2515 ^b^	3092	14.76 ^b^	29.2 ^b^	49.6 ^b^	63.0 ^b^	66.7 ^b^	70.8 ^a^	
80	84		243.0 ^a^	948 ^a^	1692 ^a^	2254 ^a^	2761 ^ab^	3113 ^ab^	3611	20.13 ^a^	50.3 ^a^	74.4 ^a^	80.3 ^ab^	72.4 ^ab^	50.3 ^a^	
80	92		252.9 ^a^	961 ^a^	1702 ^a^	2319 ^a^	2912 ^ab^	3437 ^a^	3826	21.14 ^a^	50.6 ^a^	74.1 ^a^	88.1 ^ab^	84.8 ^ab^	75.0 ^a^	
80	100		242.1 ^a^	929 ^a^	1642 ^a^	2196 ^a^	2828 ^ab^	3345 ^a^	3735	19.99 ^a^	49.1 ^a^	71.3 ^a^	79.2 ^ab^	87.7 ^ab^	68.5 ^a^	
90	84		237.6 ^a^	926 ^a^	1632 ^a^	2156 ^a^	2439 ^bc^	2842 ^ab^	4040	19.51 ^a^	49.2 ^a^	70.6 ^a^	74.8 ^ab^	69.9 ^ab^	74.3 ^a^	
90	92		252.8 ^a^	975 ^a^	1785 ^a^	2396 ^a^	2986 ^ab^	3368 ^a^	3862	21.09 ^a^	51.6 ^a^	81.0 ^a^	87.3 ^ab^	84.3 ^ab^	54.6 ^a^	
90	100		254.2 ^a^	998 ^a^	1769 ^a^	2311 ^a^	2842 ^ab^	3181 ^ab^	3876	21.26 ^a^	53.1 ^a^	77.1 ^a^	77.3 ^ab^	77.4 ^ab^	48.3 ^a^	
100	84		252.9 ^a^	1034 ^a^	1890 ^a^	2584 ^a^	3301 ^a^	3636 ^a^	3892	21.02 ^a^	55.8 ^a^	85.6 ^a^	99.1 ^a^	96.3 ^a^	60.8 ^a^	
100	92		258.0 ^a^	1050 ^a^	1828 ^a^	2388 ^a^	2887 ^ab^	3287 ^ab^	3581	21.53 ^a^	56.6 ^a^	77.8 ^a^	80.1 ^ab^	71.2 ^ab^	52.2 ^a^	
100	100		242.7 ^a^	940 ^a^	1646 ^a^	2203 ^a^	2726 ^ab^	3161 ^ab^	3625	20.15 ^a^	49.8 ^a^	70.6 ^a^	79.6 ^ab^	77.3 ^ab^	61.3 ^a^	
SEM			7.51	43.90	82.10	109.70	137.10	147.10	139.20	0.73	2.68	4.23	5.56	5.43	6.37	
*p*-value																
AA		0.82	<0.01	<0.01	<0.01	<0.01	0.01	0.08	<0.01	<0.01	<0.01	<0.01	0.52	0.64	0.02	<0.01
AME		0.21	0.01	<0.01	<0.01	<0.01	0.01	0.01	0.05	0.01	0.01	<0.01	0.01	0.43	0.83	0.56
AA × AME		0.36	<0.01	0.01	0.01	0.01	<0.01	<0.01	0.25	<0.01	0.01	0.02	0.02	<0.01	0.04	0.62

^a–c^ Means in a column not sharing a common superscript were different (*p* < 0.05). ^1^ Tukey’s test was not able to separate treatment means of BWG during d 48–55. ^2^ SEM = standard error of mean.

**Table 10:** Daily feed intake of Ross 708 broilers fed 12 diets from d 0 to 64.

Treatment		Daily Feed Intake (g/day)												
AA (%)	AME (%)	D0–10	D10–24	D24–34	D34–41	D41–48	D48–55	D55–64	D0–24	D0–34	D0–41	D0–48	D0–55	D0–64
70		22.65 ^b^	70.4	115.0	159.1	176.3	176.9	191.4 ^a^	50.5	69.5	84.8	98.1	108.3	119.7
80		25.44 ^a^	81.6	125.8	163.9	173.5	164.8	165.8 ^ab^	58.2	78.1	92.7	104.2	112.0	119.5
90		24.93 ^a^	82.3	130.8	161.5	166.2	155.0	169.7 ^ab^	58.4	79.7	93.7	102.2	107.5	113.9
100		25.27 ^a^	83.1	127.7	167.9	177.7	159.2	152.5 ^b^	59.0	79.2	94.3	106.9	113.6	119.4
SEM ^1^		0.59	2.05	3.73	4.33	5.22	6.09	6.71	1.41	1.91	2.16	2.53	2.84	3.18
	84	25.41 ^a^	82.5	131.0	171.5	184.4	170.0	186.2 ^a^	58.7	80.0	95.6	107.3	114.3	122.8
	92	25.30 ^a^	82.3	129.6	168.9	176.8	167.7	167.7 ^ab^	58.6	79.4	94.7	106.7	114.2	121.7
	100	23.00 ^b^	73.3	113.9	148.9	159.0	154.2	155.6 ^b^	52.3	70.4	83.8	94.6	102.5	110.0
	SEM	0.51	1.77	3.23	3.75	4.52	5.27	5.81	1.22	1.65	1.87	2.19	2.46	2.76
70	84		79.0 ^a^	129.9 ^a^	179.2 ^a^	199.8 ^a^			56.5 ^a^	78.1 ^a^	95.4 ^a^	110.6 ^a^	121.0 ^a^	133.7 ^a^
70	92		77.8 ^a^	126.4 ^a^	174.8 ^a^	191.7 ^a^			55.4 ^a^	76.3 ^a^	93.1 ^a^	107.5 ^a^	117.7 ^a^	129.3 ^a^
70	100		54.4 ^b^	88.7 ^b^	123.4 ^b^	137.3 ^b^			39.5 ^b^	54.0 ^b^	65.8 ^b^	76.3 ^b^	86.2 ^b^	96.1 ^b^
80	84		84.7 ^a^	128.4 ^a^	163.0 ^a^	167.3 ^ab^			60.3 ^a^	80.3 ^a^	94.4 ^a^	105.0 ^a^	110.7 ^a^	118.4 ^ab^
80	92		82.5 ^a^	132.4 ^a^	167.9 ^a^	178.2 ^ab^			59.0 ^a^	80.6 ^a^	95.5 ^a^	107.6 ^a^	116.0 ^a^	123.8 ^a^
80	100		77.6 ^a^	116.5 ^ab^	160.7 ^a^	174.9 ^ab^			55.4 ^a^	73.4 ^a^	88.3 ^a^	100.1 ^a^	109.2 ^ab^	116.4 ^ab^
90	84		81.4 ^a^	129.1 ^a^	160.7 ^a^	164.0 ^ab^			58.0 ^a^	78.9 ^a^	92.9 ^a^	98.2 ^ab^	101.9 ^ab^	107.0 ^ab^
90	92		82.1 ^a^	129.7 ^a^	170.1 ^a^	174.3 ^ab^			58.1 ^a^	79.2 ^a^	94.7 ^a^	106.3 ^a^	113.5 ^a^	120.3 ^ab^
90	100		83.5 ^a^	133.7 ^a^	153.7 ^ab^	160.3 ^ab^			59.0 ^a^	81.0 ^a^	93.4 ^a^	101.9 ^a^	107.0 ^ab^	114.6 ^ab^
100	84		84.9 ^a^	136.6 ^a^	183.2 ^a^	206.3 ^a^			60.0 ^a^	82.5 ^a^	99.7 ^a^	115.3 ^a^	123.5 ^a^	132.2 ^a^
100	92		86.9 ^a^	129.7 ^a^	162.8 ^a^	163.1 ^ab^			61.7 ^a^	81.7 ^a^	95.5 ^a^	105.4 ^a^	109.6 ^ab^	113.3 ^ab^
100	100		77.5 ^a^	116.8 ^ab^	157.8 ^ab^	163.5 ^ab^			55.3 ^a^	73.4 ^a^	87.8 ^a^	100.1 ^a^	107.6 ^ab^	112.8 ^ab^
SEM			3.55	6.47	7.50	9.04			2.44	3.30	3.74	4.38	4.92	5.51
*p*-value														
AA		0.01	<0.01	0.03	0.52	0.42	0.09	<0.01	<0.01	<0.01	0.01	0.10	0.40	0.60
AME		<0.01	<0.01	<0.01	<0.01	<0.01	0.16	<0.01	<0.01	<0.01	<0.01	<0.01	<0.01	0.01
AA × AME		0.05	0.01	0.02	<0.01	<0.01	0.06	0.46	0.01	0.01	<0.01	<0.01	<0.01	0.01

^a,b^ Means in a column not sharing a common superscript were different (*p* < 0.05). ^1^ SEM = standard error of mean.

**Table 11:** Feed conversion ratio of Ross 708 broilers fed 12 diets from 0 to 64 d of age.

Treatment		FCR												
AA (%)	AME (%)	D0–10	D10–24	D24–34	D34–41	D41–48	D48–55	D55–64	D0–24	D0–34	D0–41	D0–48	D0–55	D0–64
70		1.26	1.80 ^a^	1.79	1.98	2.14	2.44 ^b^	2.51 ^b^	1.66 ^a^	1.72 ^a^	1.79 ^a^	1.87	1.96	2.05
80		1.25	1.64 ^b^	1.72	2.02	2.16	2.65 ^ab^	3.09 ^ab^	1.55 ^b^	1.62 ^ab^	1.72 ^ab^	1.80	1.90	2.02
90		1.21	1.63 ^b^	1.72	2.05	2.23	2.94 ^a^	3.26 ^a^	1.53 ^b^	1.61 ^b^	1.72 ^b^	1.82	1.93	2.11
100		1.21	1.55 ^b^	1.65	1.98	2.23	2.91 ^a^	3.49 ^a^	1.47 ^b^	1.55 ^b^	1.65 ^b^	1.76	1.87	2.04
SEM ^1^		0.02	0.03	0.04	0.05	0.06	0.12	0.15	0.03	0.03	0.03	0.03	0.03	0.03
	84	1.27	1.68	1.77	2.04	2.29 ^a^	2.83	3.27	1.58	1.66	1.76	1.86	1.97 ^a^	2.15 ^a^
	92	1.22	1.62	1.69	1.98	2.22 ^ab^	2.74	2.92	1.53	1.60	1.70	1.79	1.89 ^ab^	2.01 ^b^
	100	1.21	1.65	1.70	2.01	2.06 ^b^	2.64	3.08	1.55	1.61	1.71	1.78	1.88 ^b^	2.01 ^b^
	SEM	0.01	0.03	0.03	0.05	0.05	0.11	0.13	0.02	0.02	0.02	0.02	0.02	0.02
70	84	1.27 ^ab^										1.92 ^ab^		
70	92	1.25 ^ab^										1.83 ^ab^		
70	100	1.27 ^ab^										1.86 ^ab^		
80	84	1.29 ^a^										1.86 ^ab^		
80	92	1.23 ^ab^										1.81 ^ab^		
80	100	1.21 ^ab^										1.72 ^ab^		
90	84	1.30 ^a^										1.97 ^a^		
90	92	1.17 ^b^										1.74 ^ab^		
90	100	1.17 ^b^										1.75 ^ab^		
100	84	1.20 ^ab^										1.70 ^b^		
100	92	1.22 ^ab^										1.79 ^ab^		
100	100	1.21 ^ab^										1.80 ^ab^		
SEM		0.03										0.05		
*p*-value														
AA		0.04	<0.01	0.07	0.78	0.67	0.02	0.01	<0.01	<0.01	0.01	0.06	0.20	0.15
AME		0.01	0.27	0.18	0.64	0.01	0.37	0.17	0.25	0.14	0.15	0.05	0.04	<0.01
AA × AME		0.03	0.20	0.06	0.33	0.26	0.40	0.61	0.25	0.14	0.16	0.03	0.20	0.23

^a,b^ Means in a column not sharing a common superscript were different (*p* < 0.05). ^1^ SEM = standard error of mean.

**Table 12:** Mortality (dead birds/total birds) of Ross 708 broilers from 0 to 64 d of age.

Treatment (%)		Mortality (%)												
AA	AME	D0–10	D10–24	D24–34	D34–41	D41–48	D48–55	D55–64	D0–24	D0–34	D0–41	D0–48	D0–55	D0–64
70		0.93	1.39	0.00	0.93	0.00	0.00	0.93	2.31	2.31	3.24	3.24	3.24	4.17
80		0.93	0.46	0.00	0.00	0.46	0.93	3.70	1.39	1.39	1.39	1.85	2.78	6.48
90		0.46	0.46	0.00	0.00	0.00	1.85	0.46	0.93	0.93	0.93	0.93	2.78	3.24
100		0.46	0.93	0.00	0.00	1.39	0.46	2.31	1.39	1.39	1.39	2.78	3.24	5.56
SEM ^1^		0.52	0.70	0.00	0.33	0.40	0.58	0.90	0.81	0.81	0.84	0.96	1.11	1.40
	84	0.35	1.04	0.00	0.35	0.69	0.35	2.08	1.39	1.39	1.74	2.43	2.78	4.86
	92	1.04	0.69	0.00	0.00	0.69	0.35	1.04	1.74	1.74	1.74	2.43	2.78	3.82
	100	0.69	0.69	0.00	0.35	0.00	1.74	2.43	1.39	1.39	1.74	1.74	3.47	5.90
	SEM	0.45	0.60	0.00	0.29	0.35	0.51	0.78	0.71	0.71	0.73	0.83	0.96	1.21
*p*-value														
AA		0.95	0.73	1.00	0.11	0.14	0.15	0.05	0.71	0.71	0.26	0.34	0.98	0.33
AME		0.45	0.83	1.00	0.67	0.14	0.09	0.25	0.94	0.94	0.99	0.77	0.86	0.34
AA × AME		0.31	0.22	1.00	0.80	0.12	0.42	0.15	0.23	0.23	0.25	0.08	0.09	0.74

^1^ SEM = standard error of mean.

**Table 13:** Feed cost and economic return analysis of Cobb 700 and Ross 708 broilers fed 12 diets at 41 and 55 d of age.

		Cobb 700						Ross 708					
Treatment		Feed Cost/BW (USD/kg)		Gross Margin Return/Bird (USD)		Gross Margin Return/BW (USD/kg)		Feed Cost/BW (USD/kg)		Gross Margin Return/Bird (USD)		Gross Margin Return/BW (USD/kg)	
AA (%)	AME (%)	D41	D55	D41	D55 ^1^	D41	D55	D41	D55	D41	D55	D41	D55
70		0.426	0.474 ^b^	2.88 ^b^	3.99	1.31	1.26 ^a^	0.415 ^b^	0.453 ^b^	2.64	3.98	1.32 ^a^	1.28 ^a^
80		0.433	0.478 ^b^	3.04 ^ab^	4.17	1.30	1.26 ^a^	0.417 ^b^	0.458 ^b^	2.98	4.20	1.32 ^a^	1.28 ^a^
90		0.433	0.483 ^ab^	3.19 ^a^	4.24	1.30	1.25 ^ab^	0.437 ^ab^	0.483 ^a^	2.97	3.93	1.30 ^ab^	1.25 ^b^
100		0.446	0.506 ^a^	3.21 ^a^	4.22	1.29	1.23 ^b^	0.442 ^a^	0.497 ^a^	3.10	4.20	1.29 ^b^	1.24 ^b^
SEM ^2^		<0.01	0.01	0.07	0.11	<0.01	0.01	0.01	0.01	0.09	0.12	0.01	0.01
	84	0.427 ^b^	0.478 ^b^	3.11	4.15	1.31 ^a^	1.26 ^a^	0.414 ^b^	0.457 ^b^	3.03	4.18	1.32 ^a^	1.28 ^a^
	92	0.424 ^b^	0.473 ^b^	3.14	4.22	1.31 ^a^	1.26 ^a^	0.413 ^b^	0.458 ^b^	3.10	4.31	1.32 ^a^	1.28 ^a^
	100	0.453 ^a^	0.505 ^a^	2.98	4.09	1.28 ^b^	1.23 ^b^	0.457 ^a^	0.502 ^a^	2.63	3.74	1.28 ^b^	1.23 ^b^
	SEM	<0.01	0.01	0.06	0.09	<0.01	0.01	0.01	0.01	0.08	0.10	0.01	0.01
70	84				4.18 ^a^					2.88 ^a^	4.34 ^a^		
70	92				4.09 ^a^					3.05 ^a^	4.40 ^a^		
70	100				3.71 ^a^					1.98 ^b^	3.19 ^b^		
80	84				4.09 ^a^					3.00 ^a^	3.99 ^ab^		
80	92				4.12 ^a^					3.08 ^a^	4.44 ^a^		
80	100				4.32 ^a^					2.85 ^a^	4.17 ^ab^		
90	84				3.79 ^a^					2.81 ^a^	3.63 ^ab^		
90	92				4.63 ^a^					3.17 ^a^	4.28 ^a^		
90	100				4.29 ^a^					2.94 ^a^	3.89 ^ab^		
100	84				4.56 ^a^					3.44 ^a^	4.75 ^a^		
100	92				4.04 ^a^					3.10 ^a^	4.12 ^ab^		
100	100				4.05 ^a^					2.76 ^a^	3.72 ^ab^		
SEM					0.18					0.16	0.21		
*p*-value													
AA		0.06	<0.01	<0.01	0.37	0.06	<0.01	0.01	<0.01	0.01	0.18	0.01	<0.01
AME		<0.01	<0.01	0.12	0.63	<0.01	<0.01	<0.01	<0.01	<0.01	<0.01	<0.01	<0.01
AA × AME		0.80	0.94	0.11	0.01	0.80	0.94	0.12	0.06	0.01	<0.01	0.11	0.06

^a,b^ Means in a column not sharing a common superscript were different (*p* < 0.05). ^1^ Tukey’s test was not able to separate treatment means of gross margins for Cobb 700 broilers at d 55. ^2^ SEM = standard error of mean.

## Data Availability

Data are contained within the article.

## Abbreviations

The following abbreviations are used in this manuscript:

AAs	Amino acids
AME	Apparent metabolizable energy
FCR	Feed conversion ratio
DBWG	Daily body weight gain
DFI	Daily feed intake
GMR	Gross margin return
